# Meckel’s diverticulum unmasked: an uncommon culprit of gastrointestinal bleeding in the young

**DOI:** 10.11604/pamj.2025.50.17.46145

**Published:** 2025-01-08

**Authors:** Shubham Patil, Sheetal Asutkar

**Affiliations:** 1 Department of Shalyatantra, Mahatma Gandhi Ayurved College Hospital and Research Centre, Salod (H), Datta Meghe Institute of Higher Education, Wardha, India

**Keywords:** Meckel’s diverticulum, gastrointestinal bleeding, ectopic gastric mucosa

## Image in medicine

Meckel´s diverticulum (MD), a congenital anomaly resulting from incomplete obliteration of the vitelline duct, often remains asymptomatic but can present with complications. Clinical manifestations include gastrointestinal bleeding, obstruction, and inflammation, posing diagnostic challenges. We present the case of a 28-year-old male with intermittent episodes of melena and vague abdominal pain over three months. The patient had no significant past medical or surgical history. He was hemodynamically stable on examination with mild tenderness in the lower abdomen. Laboratory tests revealed iron-deficiency anemia (hemoglobin 9.6 g/dL) and normal inflammatory markers. Stool occult blood was positive. Initial imaging with abdominal ultrasound was inconclusive, while a technetium-99m pertechnetate scan (Meckel´s scan) demonstrated a focal area of increased uptake in the lower ileum, suggestive of ectopic gastric mucosa within MD. Open exploration confirmed a 4cm Meckel´s diverticulum located 60cm from the ileocecal valve, with evidence of mucosal ulceration. The patient underwent successful surgery. Histopathology confirmed heterotopic gastric mucosa and chronic inflammation without malignancy. The patient´s symptoms resolved postoperatively, with normalization of hemoglobin at three-month follow-up. Meckel´s diverticulum should be considered in young patients presenting with unexplained gastrointestinal bleeding or recurrent abdominal pain. Early recognition and surgical intervention yield excellent outcomes.

**Figure 1:**
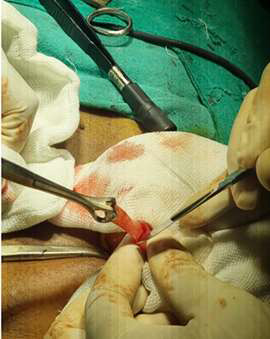
operative view of Meckel's diverticulum: a 4cm outpouching in the ileum with mucosal ulceration

